# Hydrophobic and Hydrophilic Au and Ag Nanoparticles. Breakthroughs and Perspectives

**DOI:** 10.3390/nano8010011

**Published:** 2017-12-27

**Authors:** Ilaria Fratoddi

**Affiliations:** Department of Chemistry, Sapienza University of Rome, P.le A. Moro 5, 00185 Rome, Italy; ilaria.fratoddi@uniroma1.it; Tel.: +39-06-4991-3182

**Keywords:** gold nanoparticles, silver nanoparticles, thiol ligands, metal nanoparticles networks, nnaomedicine applications, optoelectronics applications

## Abstract

This review provides a broad look on the recent investigations on the synthesis, characterization and physico-chemical properties of noble metal nanoparticles, mainly gold and silver nanoparticles, stabilized with ligands of different chemical nature. A comprehensive review of the available literature in this field may be far too large and only some selected representative examples will be reported here, together with some recent achievements from our group, that will be discussed in more detail. Many efforts in finding synthetic routes have been performed so far to achieve metal nanoparticles with well-defined size, morphology and stability in different environments, to match the large variety of applications that can be foreseen for these materials. In particular, the synthesis and stabilization of gold and silver nanoparticles together with their properties in different emerging fields of nanomedicine, optics and sensors are reviewed and briefly commented.

## 1. Introduction

Noble metal nanoparticles, whose average size falls in the range from some nanometers to micrometers, are considered, due their unusual optical properties, their size-dependent electrochemistry and their chemical stability [1], one of the leading materials in highly active fields such as catalysis [2,3], optoelectronics [4] and biosensors [5], including drug and gene delivery [6] and cancer treatment [7,8]. 

In particular, in recent years, the use of gold nanoparticles (AuNPs) in biomedicine garnered considerable attention for the potential to facilitate both the diagnosis and the treatment of cancer through their peculiar chemical and physical characteristics. One of the main properties that allows these particles to be employed in different biomedical applications is the scattering and absorption of light at resonant wavelengths. This phenomenon is known as due to the excitation of plasmon oscillations (Surface Plasmon Resonance, SPR). The resonant wavelength depends on the size, shape and geometry of the nanostructures, thus providing important information and making them the model system of choice in a wide range of biomedical applications. For example, the study of interactions of gold nanoparticles with biomolecules and cells provides new tools for the diagnosis and treatment of cancer (theranostic) and drug delivery [9,10], in particular, in computed tomography (CT) imaging and in photothermal therapy (PTT) [11,12].

### Synthetic Routes for Au and Ag Nanoparticles

Gold nanoparticles can be stabilized by many different ligands, whose chemical nature is generally chosen on the basis of the specific technological applications that are foreseen. 

Among the others, the most common method to synthesize AuNPs, is the liquid phase [13], because of its feasibility and wide use. The pioneering synthesis of gold nanoparticles has been developed many years ago with the so-called citrate route by Turkevich et al. [14]. This method involves gold chloride and sodium citrate as a reducing and stabilizing agent, respectively and water as a solvent (citrate reduction method). 

**Figure 1:**
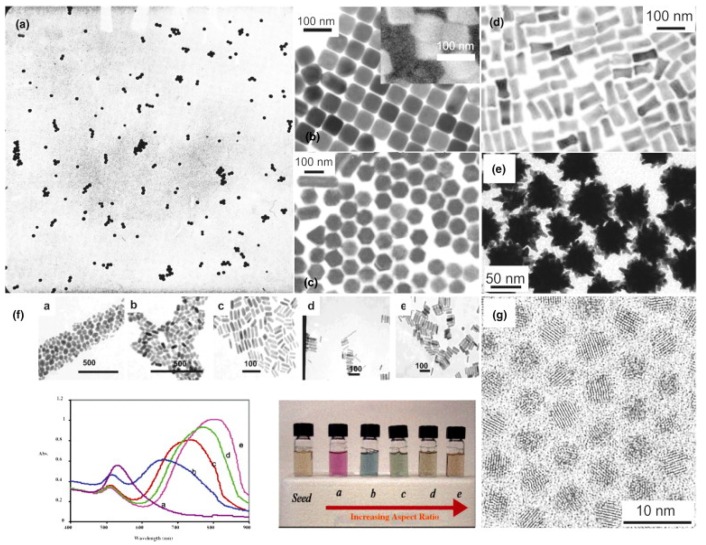
(**a**) Electron micrograph of a gold sol prepared by the citrate route; (**b**–**e**) Transmission electron microscopy (TEM) images of gold nanoparticles with different shapes; (**f**) TEM images of gold nanoparticles with increasing aspect ratios from (**a**) to (**e**) and corresponding absorption spectra and photograph of the dispersions; (**g**) High-resolution TEM image of CdSe nanocrystals. Reproduced with permission from [1]. Copyright Elsevier, 2013.

Since then, many other approaches have been proposed, performed in specific organic solvents or in the presence of different types of surfactants and other reducing agents. One of the most popular is the so-called two phases route of Shiffrin-Brust [15] a method that exploits thiol ligands that strongly bind to gold, due to the soft character of both S and Au. This method is particularly suitable to a fine tuning of the nanoparticle morphology. A variety of nano gold structures obtained by different synthesis methods is shown in Figure 1, where nanospheres, nanocubes, hexagonal shapes, nanostars and nanorods are depicted. 

More recently, many other groups have explored different methods in order to control size distribution. Among them, Natau et al. [16] proposed a modification of the Frens synthesis and Bustus et al. [17] obtained monodisperse particles through kinetically controlled seed growth.

The functionalization of AuNPs can be achieved in situ during the synthesis or through ligand-exchange reactions [18]. Depending on the chemical structure of the ligand bonded to the metal surface, the stabilization can be of steric or of electrostatic nature. In the first case, the ligands are polymers or bulky molecules, which avoid the agglomeration by steric hindrance, in the latter case the ligands are charged species, such as anions and molecules containing carboxyl groups, which produce a double electric layer that, in turn, induces coulombic repulsions among nanoparticles. 

In the last few years, a great number of ligands have been used for the stabilization of gold (and silver nanoparticles, too). Among them, we mention alcohols [19], phosphines [20], amines [21] and amino acids [22]. 

However, one of the most convenient approaches that allows an accurate control on composition, shape and narrow size distribution consists of particle functionalization with thiols [23]. Indeed, thiols stabilize the gold nanoparticle surface against aggregation and their properties can be influenced by the nature of the capping agent. Extraordinary stability of the synthesized particles is attributed to alkanethiols [24,25] that form a strong covalent bond with the particle surface, improving the solubility of nanoparticles and, moreover, allowing them to be functionalized with other functional groups. 

The size control together with the size distribution is a rather delicate process, being driven by the reactivity and passivation rate of the nanoparticles [26]. For example, in the case of molar ratios S/Au < 1/4, the particle size is controlled by the reduction kinetic of the precursors (Au^3+^, Au^+^) since the available thiol concentration is less than the amount needed for the nanoparticle capping, while for ratios S/Au > 1/4, size is determined by the competition between passivation and growth of the nanoparticle (surface coverage).

**Figure 2:**
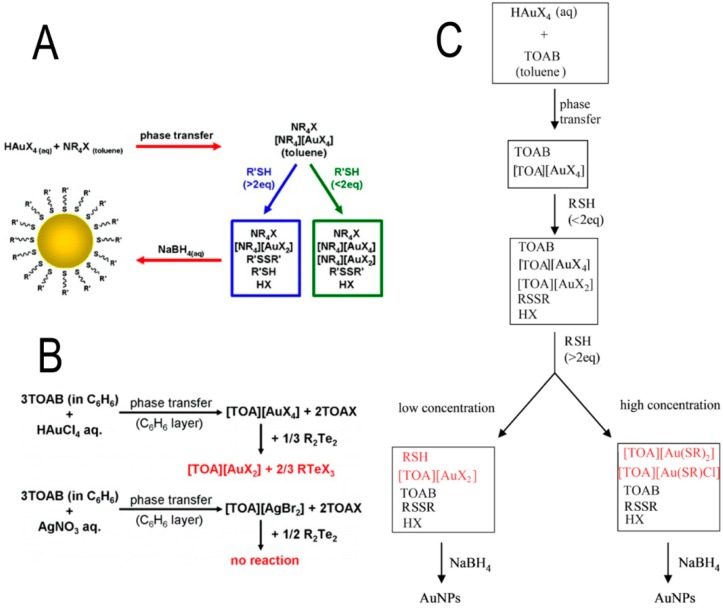
(**A**) Revised view of the two-phase Brust-Schiffrin Au Nanoparticle Synthesis; (**B**) Reaction mechanism of Au and Ag nanoparticles synthesis; (**C**) Proposed Mechanism for the Brust-Schiffrin Two-Phase Gold Nanoparticles Synthesis. Reprinted with permission from [27,28,29] respectively. Copyright American Chemical Society, 2010, 2012 and 2013.

The reaction mechanisms of the redox reaction have been recently studied in depth by different groups [27,28,29] and are schematically shown in Figure 2. 

The formation of tetra-alkyl-ammonium metal complexes is suggested to be the precursor of two-phase reaction process, while M(I) thiolates are precursors of the one-phase reactions [27]. Alternatively, the reaction intermediates of the reaction were prepared by adding (RTe)_2_ to the benzene layer of Au(III) after phase transfer with tetraoctylammonium bromide (TOAB). Gaulet et al. [27] found that (RTe)_2_ reduced Au(III) to Au(I) with no formation of a Te–Au bond [28]. A most recent work by Zhu et al. [29] assessed by nuclear magnetic resonance (NMR) spectroscopy that tetra-alkyl-ammonium gold complexes ([TOA][AuX_2_]), soluble gold thiolate ([TOA][AuSRX] and [TOA][Au(SR)_2_]) are the precursors of the Shriffin-Brust reaction and that their relative contents depend on the concentration of reactants [29]. 

The main topics related to silver nanoparticles (AgNPs) have been recently summarized by Rycenga et al. [30]. These authors in depth discuss solution-phase methods, lithographic techniques and their combinations to achieve a large number of nanostructures, such as spheres, cubes, octahedrons and triangular plates with a precisely controlled size and high uniformity. These nanostructures with sharp features are able of creating regions of high field enhancement which are responsible of the control of the plasmonic response.

Indeed, the corner sharpness, crystallinity, size and overall structure are the main features in determining the positions and the number of localized surface plasmon resonance (LSPR) modes, as well as the properties of propagating surface plasmon (PSP). 

The reduction process applied to silver is a little bit more intricate as compared to gold. As a matter of fact, in order to be extracted in a non-polar medium, an ion of a metal precursor must be electrostatically bound to a hydrophobic carrier. In the case of gold this involves the formation of an ion pair N(*n*-C_8_H_17_)_4_^+^[AuCl_4_]^−^, while for a positively charged Ag^+^ ion, such interaction becomes impossible. Nonetheless, the reduction process was widely applied in the preparation of organo-sols of metallic silver [30]. 

**Figure 3:**
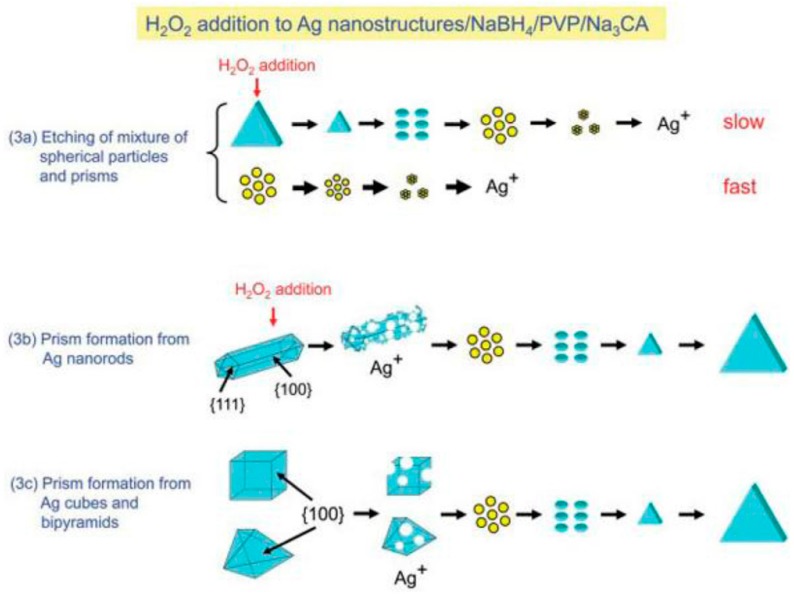
Transformation from spherical nanoparticles, nanorods, cubes, or bipyramids to triangular prisms of silver with polyvinylpirrolidone (PVP), citrate and H_2_O_2_. Growth mechanisms of Ag nanostructures prepared from mixtures of spherical nanoparticles and prisms and nanorods, cubes and bipyramids. Reprinted with permission from [35]. Copyright MDPI, 2014.

Alternative routes for the synthesis of AgNPs, which involve biological molecules as stabilizing and reducing agents, have been also proposed by Kholoud et al. [31] and by Liu et al. [32]. In a typical synthesis run of the polyol method, molecules such as ethylene glycol, 1,2-propylene glycol, or 1,5-pentanediol, serve both as a solvent and a reducing agent [33,34]. A peculiar characteristic of AgNPs is the variety of crystalline structures that can be obtained by tuning the synthetic procedures, as reported in a recent review by Zhang et al. [35] and schematically shown in Figure 3. 

Due to these morphological characteristics, AgNPs are investigated for biological, medical and antibacterial purposes [36,37]. The bactericidal property of AgNPs is probably due to their effect on cellular proteins that become inactive or to the penetration into the bacteria, thus inactivating their enzymes with the production of hydrogen peroxide that causes bacterial cell death [37]. 

AgNPs show a SPR, alike the one of AuNPs, with values in the range 400–800 nm depending on the size and shape of the nanoparticles [38]. This resonance is also useful for the development of optoelectronic devices because the tunability of the SPR peak position allows the feasibility of highly sensitive surface-enhanced Raman scattering (SERS)-active substrates for molecular identification and for SPR biosensors, sensitive to the refractive indices of surface-bonded species [38,39,40,41]. 

The main application of AgNPs can be found in the following fields; (*i*) a H_2_O_2_ responsive drug delivery system has been prepared using ultra-small (5 nm), water-stable and oxidant-prone AgNPs, therapeutically active and exploited as nanolids to cap the drug loaded nanochannels of porous silica [42]; (*ii*) by reduction of Ag ions in cross-linked dialdehyde hemicelluloses (DHC)/chitosan hydrogels, a material with strong antimicrobial activity against the model microbes *Escherichia coli* (Gram-negative) and *Staphylococcus aureus* (Gram-positive) is described in Ref. [43] by Guan et al.; (*iii*) electrospun nanofibers functionalized with AgNPs through catechol redox chemistry have been synthesized with control of the size and amount of AgNPs on the surface of nanofibers. These structures show biocompatibility, antibacterial activity in vitro and the wound healing capacity in vivo [44]; (*iv*) AgNPs immobilized on nanosilica reveal the presence of Ag_2_O on the as-prepared nanosilver surface that release Ag^+^ ions in deionized water and when exposed to a CO_2_-containing atmosphere. CO_2_ is absorbed by the host solution decreasing its pH and contributing to metallic Ag dissolution and further leaching of Ag^+^ ions [45].

## 2. Organometallic and Hydrophobic Ligands, Capping Agents for Au and Ag Nanoparticles

As above stated, the appropriate choice of ligands allows us to modify and to tailor nanoparticle properties in order to address a wide number of applications. In this section, selected recent examples of different hydrophobic molecules acting as capping agents for Au and Ag nanoparticles will be reviewed.

Based on our previous investigations on the synthesis and characterization of organometallic polymers containing Pt and Pd atoms as central metals bridging organic spacers [46,47,48] and on the study of geometry around Pt centers performed with photoelectron spectroscopy studies [49] and, finally, on some their applications as chemical sensors [50,51], our interest was recently addressed to nanoparticle assembly into more complex nanostructures [52,53,54,55]. These above stated previous studies can be considered, in a way, as the precursors of investigations on the synthesis of new hydrophobic ligands suitable as capping agents of AuNPs and AgNPs [56,57,58]. 

Among them, we mention gold nanoparticles protected by an organometallic Pd(II) thiolate synthesized on this purpose [59,60] and single-crystal gold nanoparticles obtained by applying a modified two-phase method, where a direct link between Pd(II) and Au nanoparticles through a single S bridge has been isolated.

Particle characterization (size, strain, shape and crystal structure of these functionalized nanoparticles) was carried out by a variety of experimental techniques, including a full-pattern X-ray powder diffraction analysis and high-resolution TEM and X-ray photoelectron spectroscopy (XPS) [61]. The photoluminescence spectroscopy measurements showed emission peaks at 418 and 440 nm and the exposure to gaseous NO*_x_* revealed the suitability of these nanoparticles for applications in sensor devices [60]. 

Interactions between organometallic thiol ligands and flat gold surface was also investigated, allowing us to assess the molecular orientation of these ligands and their molecular packing, being the preliminary studies for the prediction of their similar behavior on spherical nanostructures [48].

Many reports have dealt with the stabilization of gold nanoparticles with hydrophobic ligands, from the pioneering ones by Turkevich et al. and Brust et al. [14,15], to more recent ones, such as those reviewed by Heiligtag and Niederberger [1]. 

In this context, it is now well assessed that functionalization of AuNPs with hydrophobic ligands facilitates their targeted delivery to various cell types, making bio-imaging and drug delivery and other therapeutic and diagnostic applications easier. 

Polymers are a special class of gold-stabilizing agents, because these molecules are able to give core-shell nanostructures, opening a wide range of applications in bio-medicine [7,8,62].

Hydrophobic interactions have been proposed to understand solvent induced, reversible self-assembly of gold nanoparticles into tridimentional (3D) clusters with well-controlled size distribution [63]. Sánchez-Iglesias et al. [63] showed that polystyrene (PS)-stabilized spherical gold nanoparticles dispersed in tetrahydrofuran (THF) can form aggregates upon addition of water, which is a bad solvent for PS, through hydrophobic interaction and, moreover, they derived a theoretical quantitative model that accounts for nanoparticle aggregation.

A recent structure, where inorganic nanoparticles stabilized by a shell of hydrophobic organic ligands, i.e., a prototypical thiol-protected gold nanoparticle, Au_25_L_18_ (L = S(CH_2_)_2_Ph), is a particularly significant example for its property of enhancing or suppressing the natural propensity of proteins to form fibrils [64]. In this study, the authors provide a computational model of these effects on the β2-microglobulin natural fibrillation propensity and show how small nanoparticles can bind proteins to form more persistent complexes. 

In order to obtain the stabilization of citrate-capped AuNPs by the addition of amphiphilic materials (for example cetyltrimethylammonium bromide (CTAB)), two methods can be devised: (*i*) a bilayer formation of CTAB on the AuNP surfaces; (*ii*) adsorption of CTAB micelles on the AuNPs. Moreover, CTAB micelles can entrap hydrophobic molecules in their aqueous core and deliver them to the AuNP surfaces, thus allowing and favoring the study of surface-enhanced fluorescence, surface-enhanced Raman scattering and photochemical reactions of hydrophobic molecules [65].

**Figure 4:**
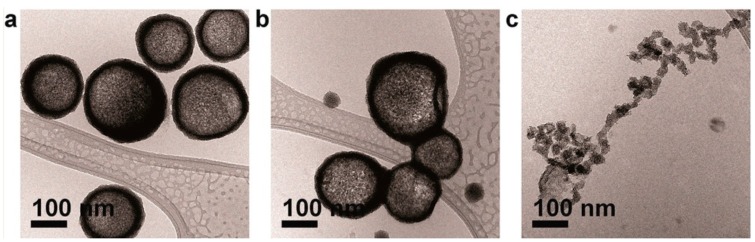
Cryo-TEM images of self-assembled AuNPs with varying surface ligand density. AuNPs were prepared with [HAuCl_4_]:[MUL] molar ratios of (**a**) 1:0.5; (**b**) 1:1 and (**c**) 1:2. The initial concentration of AuNPs was 0.3 μM for all samples. Reprinted with permission from [66]. Copyright American Chemical Society, 2015.

Recently, several strategies have been developed to combine the characteristics of vesicles and the unique physical properties of inorganic nanoparticles. Hickey et al. [66] proposed a novel approach to prepare all-nanoparticle vesicles using ligand-stabilized gold particles as a building block. All nanoparticle vesicles were synthesized by these authors by using ligand-stabilized gold particles as a building block. The hydroxyalkyl ligand rearrangement on AuNPs, which leads to increased hydroxyl group density at the nanoparticle/water interface-terminated gold nanoparticles, was found responsible of spontaneous anisotropic self-assembly of the nanoparticles into well-defined hollow vesicle-like assemblies in water, without any template. Furthermore, these authors highlight the dynamic nature of surface ligands on gold particles and demonstrate that the hydrophobic effect can be used as a versatile tool for anisotropic self-assembly of nanoparticles stabilized with 11-mercapto-1-undecanol (MUL) (Figure 4) [66]. 

An up to date review by Kobayashi et al. [67] highlights the importance of the surface engineering of AuNPs for therapeutic applications. These authors focus on three topics related to the biomedical applications, i.e., cellular membrane permeable nanoparticles, self-assembled nanoparticles and nanoparticle-based vaccines. In this review, Kobayashi et al. [67] give an extended analysis of the role of hydrophobic and hydrophilic ligands on the cellular membrane in order to encourage and favour uptake into cells. 

**Figure 5:**
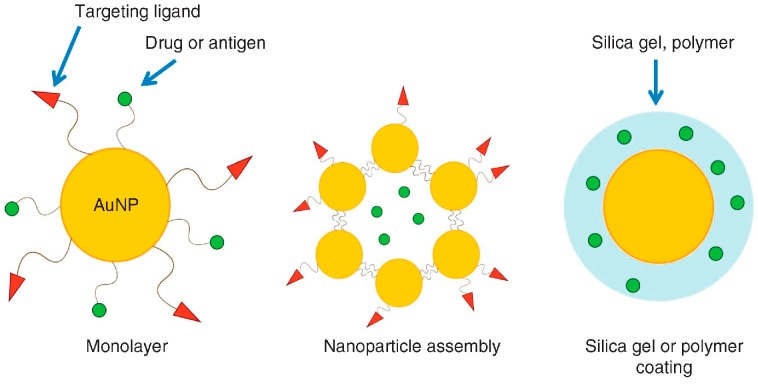
The surface modification of gold nanoparticles for the use as a drug-delivery system (DDS) carrier. Reproduced with permission from [67]. Copyright Nature Publishing Group, 2014.

Although nanoparticles require hydrophilicity for a good dispersion in water or serum in order to prevent aggregation, hydrophobicity is also required to enhance their interactions with the cell membrane. Their water dispersibility can be provided by the attachment of hydrophilic functional groups to the ligands, such as polyethylene glycol (PEG), carboxylic acids, sulfonic acids, ammonium salts or zwitterions. A delicate balance of the two properties of the ligands allows, besides dispersibility in water, cellular membrane permeability, immune responses and localization in vivo. A sketch of the various possible particle functionalizations is schematically drawn in Figure 5.

In the framework of the biological applications of gold nanoparticles, the problem of their possible toxicity has been recently reviewed [68], evidencing how different protocols employed by different researchers gave in part conflicting results, which have led to different views about their effective safety in human applications. Different factors such as shape, size, surface charge, surface coating and surface functionalization are expected to influence interactions with biological systems, at a different extent, suggesting that a critical systematization of data over the most relevant physico-chemical parameters, which govern and control toxicity, at different cellular and living systems, is highly required. 

As an example, the toxicity of AuNPs to HeLa cells [69] has been critically analyzed by inspecting the data extracted from a wide number of literature reports. We showed that, when the protocol is appropriately standardized, for example using a set of proper parameters, differently functionalized gold nanoparticles behave similarly, the different surface coatings being the critical parameter that defines the range of particle concentration where toxic effects begin. As a general trend, cell viability, starting from the initial values of 100%, progressively decreases towards lower values with the increase of the number of nanoparticles. However, this decrease occurs in different concentration regions, depending on the surface functionalization. In the case of HeLa cells, in the low concentration range, we find nanoparticles coated and stabilized by polyelectrolytes (hexadecyl-trimethyl-ammonium bromide (CTAB), phosphatidylcholine, quaternary ammines such as poly(diallydimethyl ammonium chloride) or by peptides. In the high concentration range, we find nanoparticles stabilized by tri-phenyl-phosphine and in the range in between gold nanoparticles with surface coated by a monolayer of thiols with carboxyl end groups [69].

When the ligands are peptides, a facile strategy to tailor peptide capping agents in order to improve solubility, stability and biocompatibility of AuNPs by means of the synthon reduced glutathione (GSH), has been recently proposed by Wu et al. [70]. AuNPs-GSH functionalized with tryptophan (Trp) and methionine (Met) (Au-GSH-(Trp)_2_ and Au-GSH-(Met)_2_), i.e., nanoparticles with non-polar side chains, show the greatest instability, while the incorporation of hydrophilic amino acids (histidine (His) or dansyl-labeled arginine (DanArg)) residues supports nanoparticle protection against aggregation. In this study [70] Wu et al. show once again that peptide sequence length, structure, overall charge and hydrophobic/hydrophilic balance are important factors for biological and biomedical applications. 

**Figure 6:**
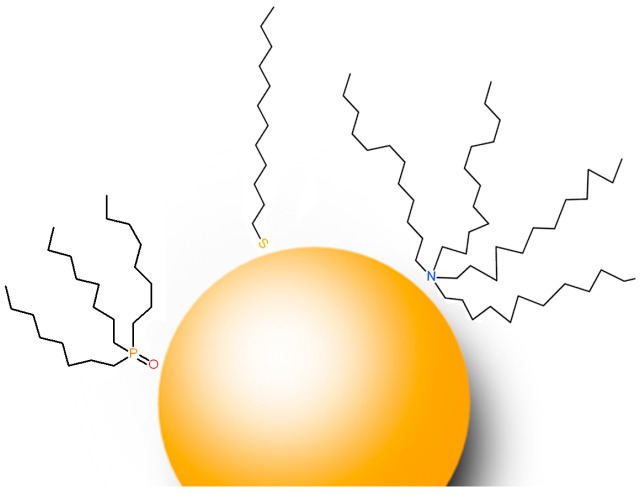
A nanoparticle idealized as a smooth sphere (5 nm in size) with different ligand molecules: trioctylphosphine oxide (TOPO), dodecanethiol (DDT) and tetraoctylammonium bromide (TOAB).

A review [71], dealing with the synthesis and properties of colloidal nanoparticles, deserves to be mentioned here. Sperling and Parak [71] refer on the proper surface functionalization of nanoparticles, which determines their interaction with the environment and a special focus is devoted to gold and semiconductor nanoparticles, such as CdSe/ZnS [71]. These authors explore, among other topics, the ligands interaction with the solvent (polar or aqueous and apolar organic solvent) and the effects of hydrophobic or hydrophilic nature of the ligands on solubility, stability and aggregation tendency are extensively examined. Examples of hydrophobic ligands interacting with gold nanoparticles are shown in Figure 6.

Some of the features taken into account in the stabilization of AuNPs with hydrophobic ligands can be also applied to the same extent to silver nanoparticles. Zhang et al. [35] highlight different synthetic methods to achieve AgNPs and present and discuss in details their main applications in biotechnology and medicine. 

AgNPs with unusual morphological structures, such as flower-like tips, have been obtained by Liu et al. [72] using CH_2_O or C_2_H_4_O as reducing agents. These tips show hexagonal-close-packed phase (HCP), besides common face-centered-cubic (FCC) phase of silver. 

Decahedral and icosahedral nanoparticles and a series of their intermediate particles which consist of a combination of two and more tetrahedra, were simply obtained by reducing AgNO_3_ in *N*,*N*-dimethylformamide (DMF) solution [73]. 

When a polymer, e.g., polyvinylpirrolidone (PVP), is used as an aiding ligand, the synthesis of triangular Ag prisms, starting from various Ag nanostructures (spheres, rods, cubes, bipyramids) was exploited using a chemical reduction method, thus allowing to produce AgNPs with surface plasmon resonance (SPR) bands at a desired wavelength [74]. 

PVP of different molecular weights can lead to high-yield of silver rodlike nanostructures, nanospheres and nanowires. In this case, the role of PVP on the shape control of silver nanocrystals is related to adsorption and steric effects depending on the PVP chain length [75]. In another example, the reduction of silver nitrate (AgNO_3_) by ascorbic acid (AsA) in aqueous poly(ethylene oxide)-poly(propylene oxide)-poly(ethylene oxide) (PEO-PPO-PEO) tri-block copolymer solutions produces the synthesis of silver crystals with various nano-scale morphologies [76]. 

Quasi-spherical Ag nanocrystals, coral-like and houseleek-like morphologies and face-centered cubic (FCC) packed micelles could be obtained with the proper addition of a copolymer. In the advanced design and practical manufacturing of metals enhanced SERS sensors, greatly branched Ag nanocrystals exhibit significant surface-enhanced Raman scattering (SERS). Silver nanowires can be subjected to chemical etching by NH_4_OH and H_2_O_2_ mixture. The surfaces of silver nanowires, synthesized by the conventional polyol method and then etched off, appear as miniature “beads on a string” features, increasing their surface roughness [77]. These nanostructured wires show enhanced active area at the tips with an increase in Raman hot spots and polarization-independent SERS signals in a scale of tens of micrometers. 

Silver nanoparticles functionalized with organic thiol allylmercaptane (AM) have been studied by us [78] by combining synchrotron radiation-based techniques, i.e., X-ray photoelectron spectroscopy (XPS) and X-ray absorption fine structure spectroscopy (XAFS). The characterizations performed on spherical like nanostructures suggest a core shell morphology of the nanoparticles (NPs) resulting in metallic Ag cores surrounded by Ag_2_S-like phase, with the external layer of AM molecules grafted to the NPs surface through Ag–S chemical bonds. 

Electron Diffraction (ED) pattern allowed to identify two different phases of single crystal corresponding to the presence of Ag face-center-cubic single-crystal symmetry, together with weak diffraction spots, in agreement with Ag_2_S cubic symmetry in Im3m space groups. 2D self-assembly networks of Ag nanoparticles could be achieved by using a peculiar ligand, i.e., an organometallic bifunctional complex (*trans*,*trans*-[CH_3_CO–S–Pt(PBu_3_)_2_(C≡C–C_6_H_4_–C_6_H_4_–C≡C)–Pt(PBu_3_)_2_–S–COCH_3_]) which was in situ deacylated to give the −SH derivative covalently bound to Ag. The structure of these nanoparticles was defined by high-resolution transmission electron microscopy (HR-TEM), selected area electron diffraction (SAED), synchrotron radiation-induced X-ray photoelectron spectroscopy (SR-XPS) and finally energy-dispersive X-ray diffraction (EDXD) analysis [79].

**Figure 7:**
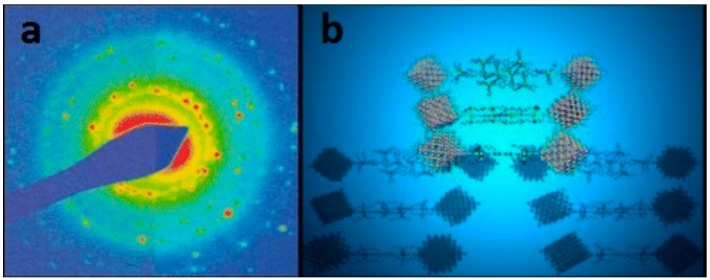
(**a**) Selected area electron diffraction (SAED) pattern of AgNPs; (**b**) draw of 2D self-assembly of AgNPs. Reproduced with permission from Ref. [79]. Copyright American Chemical Society, 2011.

As an example, in Figure 7, the characterization and imaging of Ag nanoparticles is shown. A deeper insight into the chemico-physical properties of these AgNPs is assessed by means of SR-XPS and X-ray absorption fine structure spectroscopy (XAFS) techniques [80]. 

Analogously, 2D and 3D networks of Au stabilized by the same organometallic complex, with average diameter of the nanostructures of about 3–4 nm, have been prepared and studied by our group [81], evidencing how these peculiar materials can be envisaged for optical and biological purposes. 

## 3. Hydrophilic and Amphiphilic Ligands, Capping Agents for Au and Ag Nanoparticles

Many efforts have been made in the production of AuNPs stabilized by hydrophilic or amphiphilic ligands, suitable for drug delivery or theranostic purposes. 

Among the most recent reviews on this topic, Gautier et al. [82] describe theranostic nanocarriers based on gold nanoparticles, combining both therapeutic and diagnostic properties within a single nanostructure. This review summarizes about the most addressed methods for the synthesis and the surface functionalization, providing sites for targeting ligands and for drug loading, i.e., temperature-responsive polymers, lipids, polyaminoacids, or pH-sensitive bindings that allow the release of the active molecule by the modification of the conformation of the shell, or by its degradation in the organism. 

In the same context, [83] drug delivery vehicles based on AuNPs were investigated, taking into account their diverse functionalities (for example poly(diethylene glycol) acrylate, cetyltrimethylammonium bromide (CTAB), bovine serum albumin (BSA), polyethylenimine (PEI), citrate) which allow to fulfil, by appropriate tuning of size, shape, structure and optical properties, a variety of different aims and different targets. 

In this review [83], a series of different approaches were considered, which offer opportunities in anti-cancer treatments, involving photo-thermal therapy, drug delivery, gene therapy and cell cycle regulation. Moreover, we highlight how the physiological destination of nanoparticles in vivo is still a controversial theme that needs to be further studied and understood, in view of the development of medical therapies, particularly in the case of cancer multimodal treatment. 

Gold and silver NPs, prepared with mercapto sulfonic acid (3MPS) as stabilizer (Au-3MPS, Ag-3MPS, respectively), that show interesting properties in sensors and biocidal applications, have been prepared and characterized by us by means of radiowave dielectric relaxation spectroscopy in the radiowave frequency range. This rather new approach, on the basis of *ζ*-potential measurements and d.c. electrical conductivity measurements, makes it possible to address the behavior of charged colloidal nanoparticles in the light of the standard electrokinetic model for charged particles in aqueous solution [84]. 

Hydrophilic ligands are also suitable for the synthesis of gold nanoparticles developed to improve the industrial scale up of catalytic systems. For example, AuNPs stabilized with 2-diethylaminoethanethiol hydrochloride (DEA) (or with Sodium 3-mercapto-1-propanesulfonate (3MPS)) have been prepared to build up different bio-conjugated systems that favor the immobilization of Candida Rugosa lipase (CRL) enzyme activity and stability. These structures were particularly effective in the case of Au-DEA@CRL bioconjugate, which showed a remarkable bio-catalytic performance (95% of residual lipolytic activity compared with free CRL) and a good stability in different experimental conditions (pH in the range 5–8 and temperature in the range 20–60 °C) [85,86].

A peculiar example of amphiphilic ligand for AuNPs is a bola-amphiphilic thiolate that covalently bounds to metallic nanoparticles, giving rise to spontaneous self-assembly in regular, complex structures made of ring-like domains [87]. These structures were found useful for technological development of nanostructured surfaces over macroscopic areas suitable for the integration of nanotechnology into commercial devices. 

**Figure 8:**
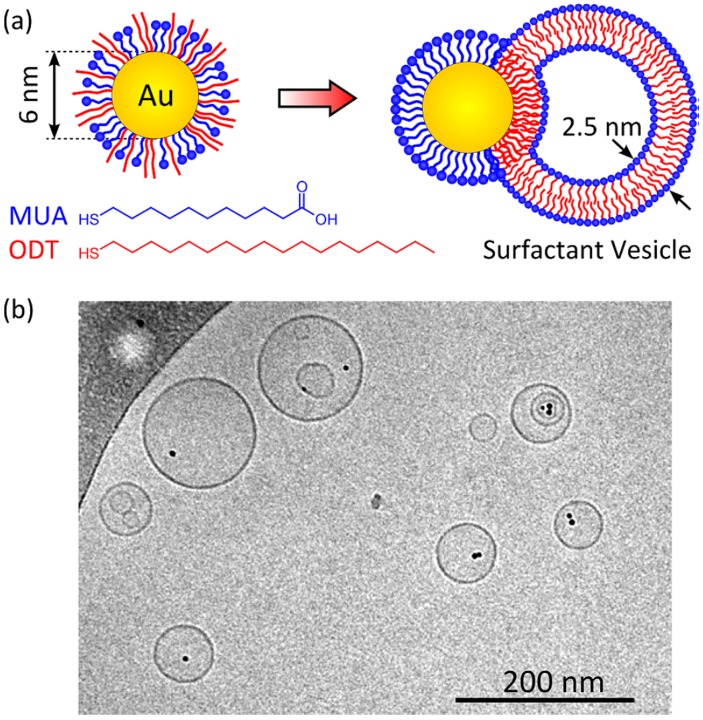
(**a**) AuNPs functionalized with mixed monolayers of ODT and deprotonated MUA are spontaneously incorporated into vesicles formed from 30:70 mixtures of cationic cetyltrimethyl ammoniumtosylate (CTAT) and anionic sodium dodecyl benzenesulfonate (SDBS) surfactants; (**b**) Cryo-TEM image showing co-localization of AuMUA/ODT NPs with surfactant vesicles in aqueous solution. The amphiphilic NPs interact with the hydrophobic core of the vesicle walls under basic conditions (pH = 11) with 100 mM added tetramethylammonium chloride (TMACl). Reproduced with permission from [88]. Copyright American Chemical Society, 2013.

Frequently, a combination of hydrophobic and hydrophilic ligands is adopted to build up nanoparticles that fulfil specific needs in biomedicine, as for example to penetrate the cell membrane and still be dissolved easily in water. For example, relatively large (6 nm in diameter) gold NPs functionalized with mixed monolayers of hydrophobic octadecanethiol (ODT) and hydrophilic mercaptoundecanoic acid (MUA) are spontaneously incorporated into the walls of surfactant vesicles (2.5 nm thick) as schematically shown in Figure 8. The formation of NP-vesicle structure is achieved simply by mixing amphiphilic AuNPs and preformed surfactant vesicles in aqueous solution. The amphiphilic NPs described in their paper by Lee et al. [88] are thought to provide a useful model system for the study of multiscale assembly processes, with the incorporation of water soluble particles even when the size of the particle greatly exceeds the layer thickness.

The self-assembly behavior is important for a wide range of applications, i.e., drug delivery carriers, exploitation of electrodes, sensors and electronic to optical devices. In particular, Janus particles, due to the presence of both hydrophilic and hydrophobic ligands on the same nanoparticle, self-assemble into vesicle or worm-like string structures. A recent example of Janus-like AuNPs is reported by Iida et al. [89], where hexa-ethylene glycol-terminated thiolate ligands with alkyl chains of different length and a hydrophobic ligand, a butyl-headed thiolate, were linked to gold nanoparticles. This investigation had the twofold objective: (*i*) a quantitative analysis of the phase segregation of the two ligands with different alkyl chain lengths toward the formation of Janus AuNPs with hydrophilic/hydrophobic faces and (*ii*) the observation of self-assembled NP structures with a Janus-type surface in water.

It is noteworthy that one-step and two-step ligand exchange synthetic methods give different aggregation behavior of the Janus-like AuNPs, estimated using Matrix-Assisted Laser Desorption/Ionization time-of-flight mass spectrometry (MALDI-TOF MS) analysis based on Cliffel’s method. By the two-step approach, large phase segregation was achieved for AuNPs of 5 nm in size, which formed assemblies of about 160 nm in diameter, while the one-step reaction produced domain, in which the two ligands formed partial domains on the surface.

Another rather interesting application of AuNPs coated with alkanethiols and mercaptoalkanoic acids is foreseen in the use of porous monolithic capillary columns designed and prepared for chromatographic separations of proteins in the mixed mode [90]. These columns are made of poly(glycidyl methacrylate-co-ethylene dimethacrylate) monolith, reacted first with cystamine and subsequently treated with TCEP (tris(2-carboxylethyl)phosphine) to cleave the di-sulfide bridges of cystamine and liberate the desired free thiol groups. AuNPs (15 nm in size) dispersion was then pumped through the monolithic column and nanoparticles were attached to the pore surface. The monolithic stationary phases allow the separation of proteins in the same column using gradient elution conditions, typical of reverse phase and ion exchange chromatographic modes, respectively.

The controlled grafting of a large number of amine-terminated histamines and short PEG chains onto a poly(isobutylene-*alt*-maleic anhydride) backbone leads to the preparation of multifunctional amphiphilic polymers which can be employed as ligands for metal nanostructures, such as nanoparticles made of a gold core [91]. Wang et al. [91] emphasize that this polymer coating can be adjusted to various metal and metal oxide surfaces, such as iron oxide and that the resulting NPs can be used to develop biologically-active platforms with potential use for drug delivery and sensing.

Likewise, core-shell gold nanoparticles, stabilized with a hydrophilic polymer, poly(3-dimethylammonium-1-propyne hydrochloride) (PDMPAHCl), show the capability of a non-covalent immobilization of bovine serum amino oxidase (BSAO), a polyamine-degrading enzyme used in the cancer treatment, trough functionalization of the surface due to the presence of aminic groups [92,93]. This bioconjugate system is pH responsive, providing an enzymatic activity up to 40% and is a promising candidate for biomedical applications to selectively generated reactive oxygen species into cancer cells. 

In this framework, it is of interest a newly designed synthetic approach described by Liu et al. [94]. These authors provide hydrophilic copolymers containing pendent thiol groups along a polyethylene glycol (PEG) methacrylate backbone by classical free radical copolymerization, that were used as multidentate ligands for AuNPs coating. These multi-dentate polymers modified AuNPs showed hydrodynamic diameters between 40 and 50 nm and high colloidal stability, protein resistance and phagocytosis by macrophages in vitro. 

AuNPs in vivo exhibit a different behavior. A typical example is given by nanoparticles coated by ligands with different length PEG fraction. In the presence of higher PEG fraction, nanoparticles show a lower accumulation in the liver, longer retention in the blood and higher uptake in the tumours, while AuNPs coated by ligands with relatively lower PEG fraction had lower accumulation in the spleen [94]. 

PEG and its derivatives are among the main polymeric ligands used to coat and stabilize AuNPs, because they are biocompatible, stable and suitable for chemical modifications to fulfil different biomedical needs. 

**Figure 9:**
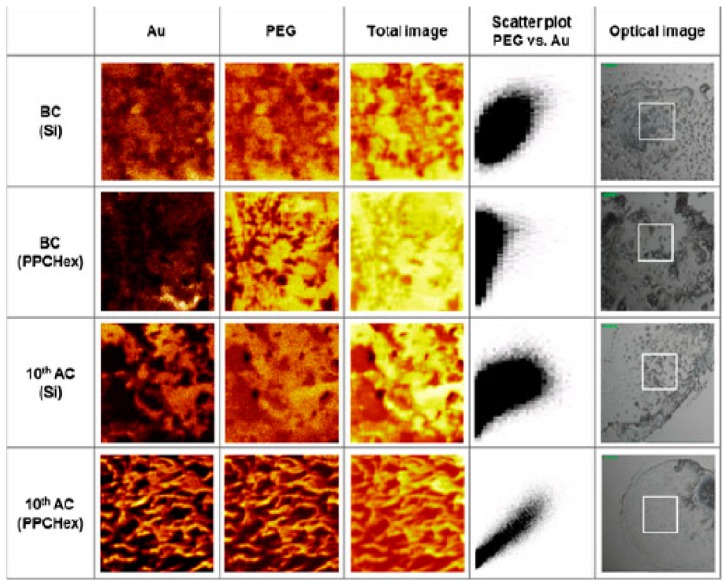
ToF-SIMS images of Au ions (Au-), mPEG-related ions (C_3_H_3_O_2_-) and total ions together with the scatter plots of Au and mPEG ion intensities, which were obtained from the BC (before centrifugation) and the AC (after 10 centrifugation) samples on a Si wafer and a PPCHex wafer. Optical image for each dried pattern is also shown with the box of analysis area. Reproduced with permission from [95]. Copyright John Wiley and Sons, 2012.

Due to the complexity of the system in different biological environments, it is then mandatory to find methods that, besides well-known UV-visable spectroscopy (UV-vis), Dynamic Light Scattering (DLS) and zeta potential characterizations, are able to study the stability of ligand-conjugated nanoparticles in suspension. The statistical analysis of time-of-flight SIMS (ToF-SIMS) imaging analysis provides more precise and quantitative results about the coexistence of the AuNPs and PEG, by means of the calculated Pearson product-moment correlation coefficient (PMCC) of the AuNPs and PEG intensities [95]. A typical example of ToF SIMS images is shown in Figure 9. Shon et al. [95] suggest that this new approach could simplify the method by which it is possible to quantitatively study the degrees of coexistence between nanoparticles and ligands, the number of free ligands and the stability of ligand-conjugated nanoparticles in suspension.

### 3.1. Ligands Coverage Assessment

Another important topic of interest in NPs characterization is the assessment of the degree of coverage, i.e., the number of ligands attached to the nanoparticle surface. Hinterwirth and co-workers [96] measured the gold-to-sulfur [Au/S] ratio for thiol stabilized nanoparticles by means of inductively coupled plasma mass spectrometry (ICP-MS) and its dependence on the nanoparticle diameter. 

**Figure 10:**
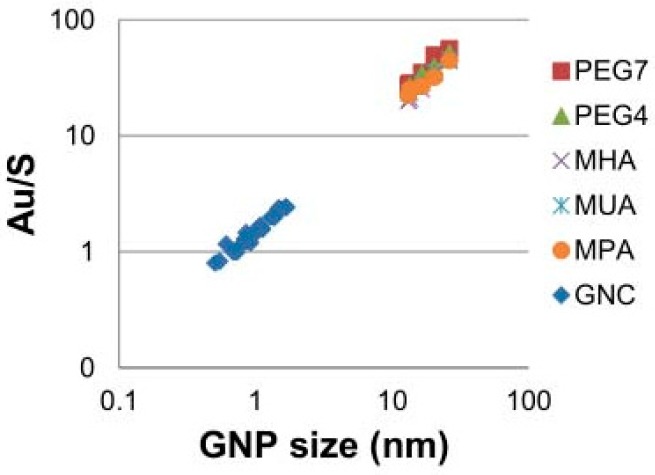
Plot of Au/S ratio as determined by ICP-MS measurements vs. AuNP (GNP) size. Reproduced with permission from [96]. Copyright American Chemical Society, 2013.

Since the average number *N*_Au/AuNP_ of gold atom per AuNP will increase with the cube of the diameter *D* and the number *N*_S/AuNP_ of sulfur atom per AuNP will increase with its square, the Au/S ratio should increase linearly with the diameter *D* according to

$$\frac{N_{\mathrm{Au}/\mathrm{AuNP}}}{N_{S/\mathrm{AuNP}}}=\frac{\varrho D}{6M_{\mathrm{Au}}K}\;\sim \;9.38\;\frac{1}{K}\;D\;\left[\mathrm{nm}\right]$$

where *K* is the maximum coverage factor and *ρ* is the density for fcc gold and *M*_Au_ is its atomic weight. This equation allows the calculation of the maximum ligand density from the slope of the plot of the Au/S ratio versus nanoparticle diameter, assuming a constant and NP-size independent maximal coverage *K*. The validity of this relationship is supported by Figure 10 which proves a linear dependency and thus a constant slope.

**Figure 11:**
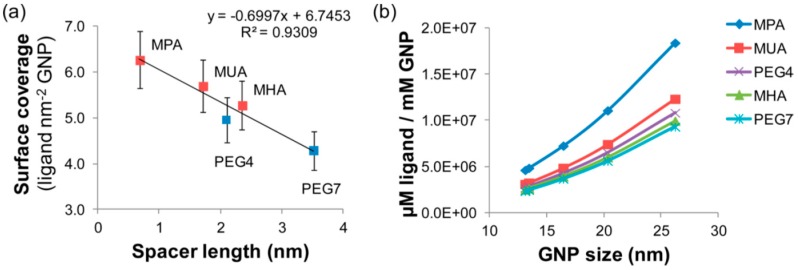
(**a**) Influence of ligand length on surface coverage (squares, red = mercapto-alkanoic acid, blue = mercapto-(PEG)ncarboxylic acid); (**b**) Total number of ligands per GNP as calculated from the results of panel a and particle size for the different types of surface modifications. (GNP is the same as AuNP). Reproduced with permission from [96]. Copyright American Chemical Society, 2013.

AuNPs were derivatized on the surface with bifunctional (lipophilic) ω-mercapto-alkanoic acids (MHA, MUA, MPA) and (hydrophilic) mercapto-poly(ethylene glycol) (PEG), (PEG7, PEG4) carboxylic acids, respectively, by self-assembling monolayer formation. The ligand density decreased with increasing ligand chain length, i.e., the surface densities ranged between 6.3 molecules nm^−2^ for the short ligand MPA (3-mercaptopropionic acid, spacer length = 0.68 nm) and 4.3 molecules nm^−2^ for the longer PEG7 ligand (spacer length = 3.52 nm). Thereby, no significant difference between lipophilic mercaptoalkanoic acid and hydrophilic mercapto-(PEG)4-carboxylic spacer was observed, indicating that steric hindrance is of more importance than other kinds of interactions. Figure 11 shows the correlation of ligand length with nanoparticle coverage and of number of ligands with particle size [96]. 

**Figure 12:**
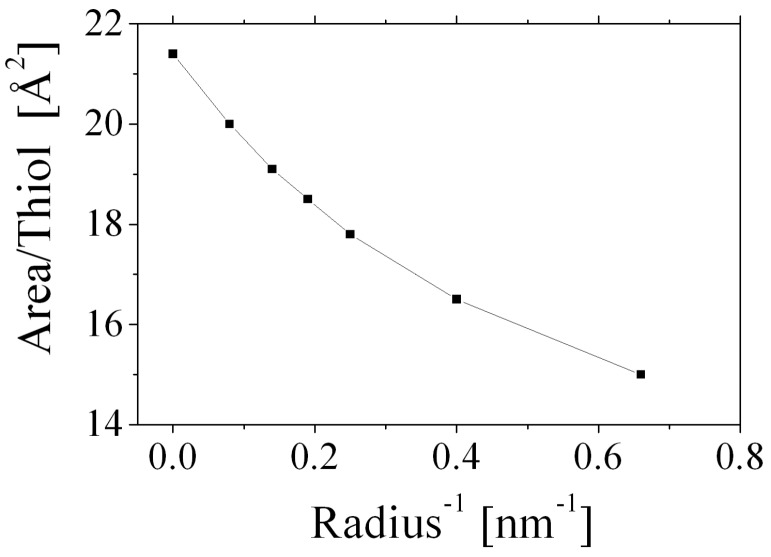
Surface area occupied by a single thiol chain as a function of the inverse radius, according the molecular mean-field theory developed by Tambasco et al. Reproduced with permission from [99]. Copyright American Chemical Society, 2008.

As a further example, in the case of self-assembled dodecanethiol monolayers on planar Au surfaces, experimental measurements showed that each surfactant molecule occupies an area of 21.4 Å^2^ on the Au surface [97]. However, each thiol occupies only 15.5 Å^2^ for a gold nanoparticle 3 nm in size [98] and the thiol coverage per unit area increases with the decreasing particle size. In Figure 12 the surface area occupied by a single thiol chain is shown as a function of the inverse particle radius, according to molecular mean-field theory [99].

Burda et al. [100] found the optimal synthesis ratios of PEG/AuNPs (size about 6 nm) to achieve stability and maximum dispersity, by using PEG 0.55, 1, 2 and 5 kDa at the PEG/AuNP ratios 2500, 700, 500 and 300, respectively. These authors report surface ligand density, hydrodynamic size, dispersity and cellular toxicity evaluation to assess cell viability and interaction with HeLa cervical cancer cells. In particular, the calculation of the ligand density was achieved via Au/S ratio considering that each ligand on the Au NPs surface carries a single S atom, while Au atoms constitute the NP core. The thermal decomposition of PEG on the AuNP surface with release of volatile sulphur leads to calculate the ligand grafting density of each AuNP from the number of AuNPs and PEG chains. 

### 3.2. Drug Delivery Applications

The behavior of AuNPs functionalized with hydrophilic thiol ligands, containing poly(ethylene)glycol groups was recently revised by Kunstmann-Olsen et al. [101] by means of environmental scanning electron microscopy (ESEM). Self-assembly in patterns was highlighted and the process was elucidated in situ by ESEM during both evaporation and condensation of the dispersant on a variety of substrates, including pre-patterned ones. It was found that attractive interactions between the substrate and AuNPs are often stronger than what expected, once the particles have been deposited. Moreover, the role of a highly water-soluble additive was investigated. Interestingly, it was found that entropy driven deposition of particles and decoration of surface features were enhanced in the presence of nickel perchlorate.

**Figure 13:**
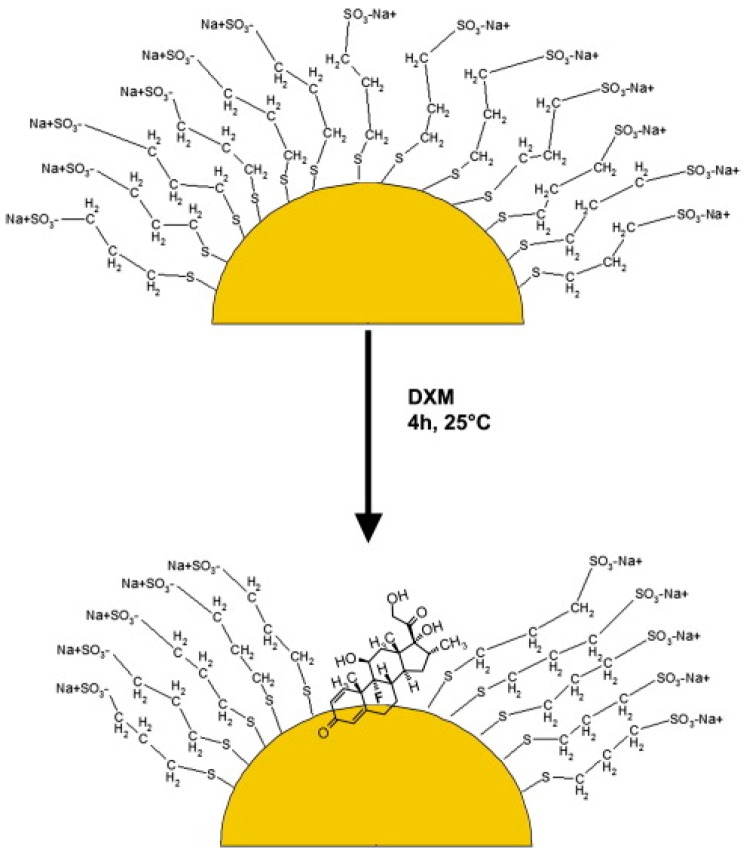
Interaction scheme between DXM and Au-3MPS. Reprinted with permission from [102]. Copyright Elsevier, 2014.

Au-3MPS nanoparticles with spherical morphology and average size of 7–10 nm (3MPS; Sodium 3-mercapto-1-propanesulfonate) have been investigated [102,103] for their high potential in biotechnological and biomedical applications, mainly for the loading (70–80%) and release (70% in five days) of water insoluble drugs, such as dexamethasone (DXM). Figure 13 shows a sketch of the Au-3MPS interaction with DXM. The number of ligands on the surface of the gold nanoparticles was estimated to be about 720, i.e., a single 3MPS thiol every 10 surface atoms. It is also noteworthy that the drug nanoparticle interaction occurs through fluorine atoms of DXM and Au(I) atoms at the nanoparticle surface, as assessed by a combined investigation on the basis of NMR and XPS studies. The 3MPS ligands provide the water solubility, while a closely packing gives room enough for the drug attachment. 

The same drug loading and release was investigated by using nanostructured polymers and copolymers (size in the range 190–500 nm) with up to 90% of drug loading for P(PA-co-AA)@DXM with 8/1 PA/AA monomer ratio (PA is phenylacetylene and AA is acrylic acid). The bioconjugate showed apoptosis inhibition of human tumor cells (HeLa) [104]. 

Alternatively, the specific recognition of cancer cells can be exploited with photo-responsive plasmonic vesicles, made up by amphiphilic AuNPs, carrying hydrophilic poly(ethylene glycol) (PEG) and photo-responsive hydrophobic poly(2-nitrobenzyl acrylate) (PNBA) [105]. 

**Figure 14:**
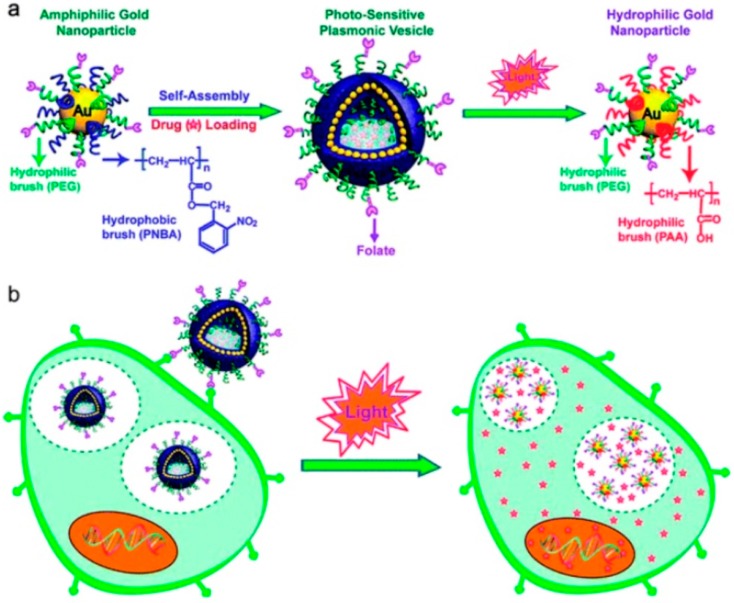
Upper panel: Schematic illustration of self-assembly of amphiphilic gold nanoparticles with mixed polymer brush coatings into plasmonic vesicles and photo-responsive destruction of the vesicles. Bottom panel: Cellular binding and photo-regulated intracellular payload release of the plasmonic vesicles. Reproduced with permission from [105]. Copyright Royal Society of Chemistry, 2013.

The plasmonic vesicles assembled from these nanoparticles exhibit optical properties and provide flexible spacers for bio-conjugation of targeting ligands to facilitate the specific recognition and imaging of cancer cells and photo-regulated drug delivery. The targeted delivery of model anticancer drug (doxorubicin) was investigated by dual-modality plasmonic and fluorescence imaging, together with toxicity studies. Figure 14 shows a typical example of action for photolabile plasmonic vesicles as multi-functional drug carriers [105].

Astruc and co-workers [106] have synthesized AuNPs stabilized by hydrophilic dendritic macromolecules, i.e., 1,2,3-triazole-containing nona-PEG branched dendrimers. The AuNPs are water soluble, have very small sizes (between 1.8 and 12 nm) and show very efficient *p*-nitrophenol reduction property [106]. 

According to the examples above stated so far, it is clear that in the field of materials for nanomedicine applications, as carriers of biomolecules, AuNPs have a paramount role. More strictly biological are examples dealing with RNA and DNA binding to AuNPs. A recent review by Rotello and coworkers [107] accounts for AuNPs as carriers of nucleic acids and small interfering RNA, (siRNA) in particular. This topic is relevant in studies related to advances in cancer therapy and genetics, where structural design of AuNPs for nucleic acid delivery vesicles is required. First, the design of AuNP-based covalent and noncovalent nucleic acid carriers is discussed, since it significantly affects cellular uptake, endosomal escape and nucleic acid release. Rotello et al. [107] emphasize how short- and long-term cytotoxicity of AuNPs is essential for their use in clinical applications. Then, targeting of these vehicles to specific organs and tissues is discussed, suggesting two different approaches, i.e., decorating the surface with specific antibodies targeted to the disease cells and grafting non-interacting functional groups (e.g., polyethylene glycol and zwitterionic entities) on the surface that avoids plasma protein adsorption, thus improving the pharmaco-kinetics and elude immune surveillance [107].

A specific example on this issue deals with the delivery of siRNA that was studied with a non-cationic bifurcated ligand (BL), possessing short hydrophobic (octyl ether) and hydrophilic (hexaethylene glycol) arms, as surface ligand for (AuNPs) [108]. Every single AuNPs (size 40 nm) stabilized by (16-mercapto-hexadecyl) trimethylammonium bromide (MTAB), immobilized 26 siRNA with electrostatic interaction and had negative zeta-potential due to siRNAs on the outermost surface. The explanation proposed by Niikura et al. [108] is that amphiphilic property should allow AuNPs to permeate the cell cytosol thorough the endosomal membrane and TEM images on HeLa cells incubated with the bioconjugate siRNA-BL/MTAB-AuNPs support this hypothesis. 

Studies concerning DNA interaction with drug molecules through bonding with AuNPs has been object of extensive research efforts. 

**Figure 15:**
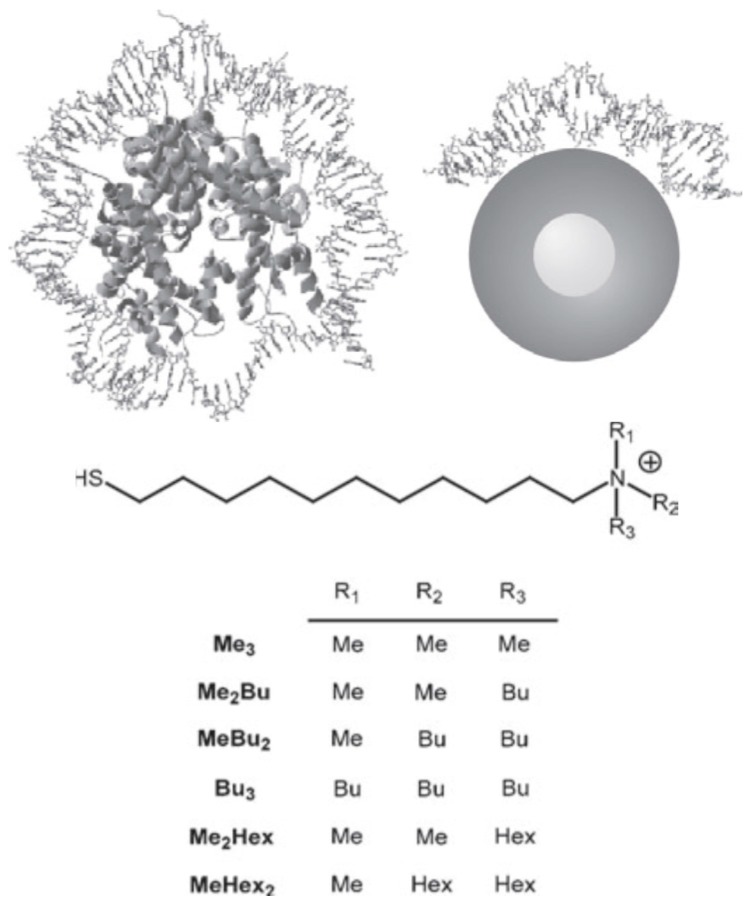
Upper panel: To scale depictions of the histone octamer and the cationic nanoparticle, both with DNA bound on the surface. The similar curvature and particle radius suggests similar modes of binding may be important. Histone tails have been removed for clarity. Bottom panel: Structure of the functionalized monolayer components. The coverage of these quaternary ammonium salt chains in the monolayer was determined to be 60 ± 10% by nuclear magnetic resonance (NMR). Reproduced with permission from [111]. Copyright John wiley and Sons, 2006.

In the past, various investigations described the aggregation induced by non-cross-linking DNA hybridization for DNA-functionalized gold nanoparticles [109] and the design of a hybridization assay based on colour changes associated with gold aggregation of single- and double-stranded oligonucleotides, that have different propensities to adsorb on gold nanoparticles was reported [110]. Analogously, these investigations dealt with the mechanism of mixed monolayer-functionalized gold nanoparticles—DNA interaction by studying a range of quaternary amines containing groups with increasing hydrophobic bulk, giving insight into the relative contributions of non-covalent forces to DNA binding (Figure 15 shows the binding mode of AuNPs with DNA). Finally, circular dichroism and fluorescence experiments showed that nanoparticle-binding causes a reversible conformational change in the DNA structure [111].

On the same issue, more recent papers deal with: (*i*) the synthesis of a model system made by a DNA-protein-effector triplex system in which the interaction between the protein and DNA can be regulated by the effector (e.g., protein or small molecule) and evidenced through the coupling with gold nanoparticles [112], (*ii*) gold nanoparticles capped with *N*-(2-mercaptopropionyl) glycine interacting with double stranded DNA investigated with a simple three-step mechanism reaction scheme [113] and *(iii*) gold nanoparticle wire sensors, promising for detecting viruses, whose detection sensitivity depends on the gold nanoparticle size, DNA concentration and DNA length [114].

### 3.3. Aunp-Based Composites

A brief outline on AuNP-based composites is hereafter mentioned. A previous work by Vossmeyer et al. [115] described the use of composite metal nano-particle/organic films for chemical sensing. In particular hydrophobic polyphenylene dendrimers linked to AuNPs were found to enhance the sensitivity to volatile organic compounds (VOCs) and to suppress undesired cross-sensitivity to humidity. AuNPs were directly synthesized in polyvinylpyrrolidone (PVP) solution by laser ablation. Furthermore, nano-fibrous composites were obtained by the electrospinning of the solution with a clean and chemically safe method [116]. 

Temperature sensitive polymer, i.e., poly(*N*-isopropylacrylamide), (Poly(NIPAM)), was covered by spiky gold nanoparticles possessing a strong and broad absorption band. These composite materials exhibited a strong and broad absorption band and photon-to-heat conversion property, besides fast reversible structural diameter changes upon exposure to a broad band light. In this way, the composite is thus suitable for the development of photo-thermally triggered carrier systems [117].

A novelty in composite exploitation is the use of liquid crystals as host matrix. In this framework, a recent review by Choudhary et al. [118] highlights composites made by AuNPs that were evenly dispersed into liquid crystals, addressing two important features, i.e., tuning of AuNPs properties by liquid crystals and vice versa [118]. 

Our research also has been applied to the development of composites. For example, polyaniline (PANI) hosted AuNPs, giving composites with different morphologies, ranging from amorphous to sponge-like and spherical shapes. These materials were used for the development of chemical sensors with high sensitivity to ammonia (up to 10 ppm), higher than that of other VOCs or interfering analytes [119]. 

Incidentally, as far as the field of chemical sensors is concerned, it is worthy to point out the sensitivity to hydrogen of platinum nanoparticles (PtNPs, size 3–10 nm) coated with 3-mercapto-1-propanesulfonate (3MPS) as a hydrophilic capping agent, that were deposited on titania nanofibers obtained by electrospinning [120].

**Figure 16:**
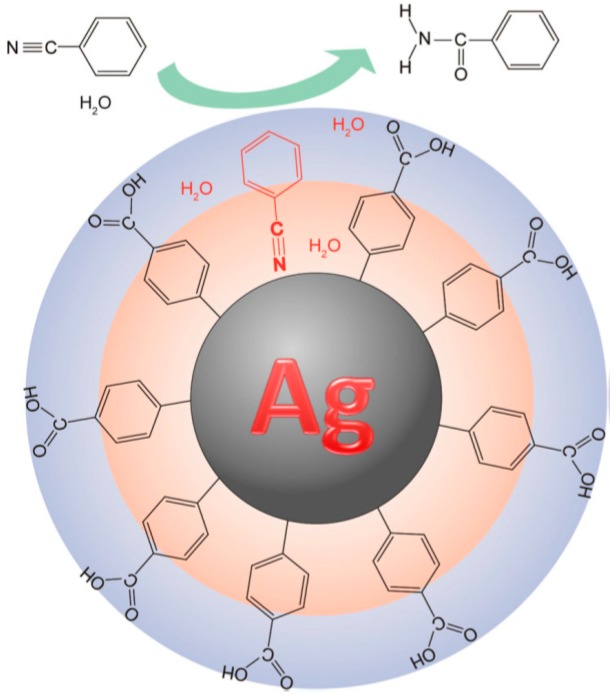
Schematic illustration of catalytic mechanism of the selective hydration of nitriles to amides using Ag NPs stabilized by hydrophilic organic ligand molecules via Ag–C covalent bond. Reproduced with permission from [121]. Copyright Springer, 2015.

Hydrophilic capping agents have been investigated to achieve an important property for AgNPs in bio-medical applications and catalysis, i.e., water solubility or dispersion. For example, Kawai and co-workers [121] prepared water-dispersible AgNPs with a specific surface and interface that catalysed the selective hydration of nitriles to amides in water. AgNPs were stabilized by aromatic ligand molecules connected with silver-carbon covalent bonds and the effect of surface surrounding NPs on catalytic activity was examined by evaluating the catalytic activity of silver NPs with hydrophobic/hydrophilic double layers [121]. A scheme of the catalytic route is drawn in Figure 16. 

The effective activity of Ag against bacteria, viruses and other eukaryotic microorganisms is well known and some examples have been already cited in this review talking of hydrophobic ligands.

An interesting study on the antibacterial activity of AgNPs stabilized with polyhexamethylene biguanide, casein protein and sodium citrate was carried out by Ahmed et al. [122]. The AgNPs were tested against different Gram-positive and Gram-negative waterborne bacteria, 8 different bacterial isolates at concentrations varying from 26 to about 77 µg/mL and nanoparticles with size 3–8 nm, prepared by casein stabilization method.

The antibacterial efficacy of AgNPs was also tested for oxidant prone AgNPs as nanolids to clog the drug encapsulated nanopores of silica for the development of an oxidant responsive drug delivery system [42]. A water-soluble and highly biocompatible triblock copolymer F127, (PEO)_106_(PPO)_70_(PEO)_106_, was the stabilizing agent leading to 5 nm sized nanoparticles that, upon exposing to H_2_O_2_, through dissolution-accompanied aggregation of Ag nanolids, allow to run free the encapsulated therapeutics from silica channels. 

Antimicrobial activity against the model microbes *Escherichia coli* (Gram-negative) and *Staphylococcus aureus* (Gram-positive) has been achieved also with hemicelluloses-based hydrogel coated with Ag nanoparticles. The hydrogel network was prepared by the Schiff base reaction between the amino groups of chitosan chains and the aldehyde groups of dialdehyde hemicelluloses [DHC] [43]. 

It is interesting to mention another bioinspired application. Wound healing can be enhanced by electrospun nanofibers that contain antibacterial silver nanoparticles. The nanofibers are made by a mussel-inspired copolymer, poly(dopamine methacrylamide-co-methyl methacrylate) (MADO), that uniformly attaches the AgNP at its surface, through the catecholic moiety of dopamine methacrylamide in polymeric backbone.

In vitro and in vivo tests assessed the bioavailability and antimicrobial activity of MADO-AgNPs [44]. Although AgNPs are widely used in commercialized formulas for health care, however their exposure to humans might increase some risk for public health. A work by Pratsinis, Fujiwara et al. [45] deals with the study of the release of Ag ions in a nanosilver suspension exposed to a CO_2_ containing atmosphere, like ambient air.

AgNPs (size 7–30 nm) immobilized or supported on nanostructured SiO_2_ (Ag/SiO_2_) and containing 50 wt % Ag were made by flame spray pyrolysis (FSP) of appropriate solutions of silver acetate and hexamethyldisiloxane. Nanosilver slowly dissolves and releases Ag^+^ ions in solution over a much longer period than the initial dissolution of any Ag_2_O layer on nanosilver, thus increasing the antibacterial efficacy, while enhancing the risk to environment by CO_2_ absorption in water suspension [45].

## 4. Conclusions

This review gives an overview of the most recent achievements on gold and silver nanoparticles, which exhibit a variety of properties and applications depending on the kind of ligands that are their capping agents. The breakthroughs and perspectives range from biotechnology, nanomedicine, sensors and catalysis. It is well known that prerequisite for every possible application is the proper surface functionalization of the nanoparticles, which determines their interaction with the environment. The functionalization affects the size, shape, solubility, stability and assembly of both AuNPs and AgNPs. Therefore, the careful and proper choice of the nanoparticles ligands is the leading feature for the achievement of the desired application. 

Hydrophobic ligands are also suitable for biomedical purposes, often in combination with hydrophilic ones, especially in the case of AuNPs, giving nanoparticles that fulfil promising theranostic properties. AgNPs stabilized with hydrophobic ligands are also widely investigated for being effective growth inhibitors against various microorganisms and then to be used in medicine as well. Catalysis is also well addressed among the perspectives for silver nanoparticles use.

Hydrophilic ligands for both Au and Ag nanostructures are preferred in case of therapeutic requests, because their water solubility allows the cells viability and circulation in blood vessels, in order to successfully reach target organs. Moreover, these ligands can be further functionalized with biomolecules such as drugs or antibodies, in order to match with the needs of fighting tumors or cancer.

Finally, the applications of AuNPs and AgNPs in sensors have been only occasionally mentioned here because the choice of ligands is less crucial for the nanoparticle performance. However, these topics are also important features that need our attention.
